# Novel Secure Group Data Exchange Protocol in Smart Home with Physical Layer Network Coding

**DOI:** 10.3390/s20041138

**Published:** 2020-02-21

**Authors:** Qiao Liu, Wenjing Zhang, Sheng Ding, Hui Li, Yong Wang

**Affiliations:** School of Cyber Engineering, Xidian University, Xi’an 710126, China; xd.zhangwenjing@gmail.com (W.Z.); shawnding.xdu@gmail.com (S.D.); lihui@mail.xidian.edu.cn (H.L.); wangyong@mail.xidian.edu.cn (Y.W.)

**Keywords:** smart home, group data exchange, physical layer security, nested lattice code

## Abstract

Smart homes have been shown to be one of the most important applications of Internet of Things (IoT); however, security issues are still the main drawback to be improved, especially facing the problem of terminal power constraint and distributed network architecture. In this paper, we propose a novel secure group data exchange protocol in smart homes with physical layer approaches which retains the benefit of key sharing needless and lightweight computation. As the core technique, nested lattice physical layer network coding is conduct in each sensor node to form a summed data at a home router. With such summed data, the untrusted home router attack and external eavesdropper attack can be resistant. Performance has been analyzed for the proposed protocol in terms of time slot cost, security resistance, and secrecy capacity. Finally, simulations have been conducted to demonstrate the theoretical analysis.

## 1. Introduction

With the continuous development of information technology, the smart era has arrived. Novel thoughts and applications have been proposed for smart phones, smart vehicles, smart grids, smart health, and so on. Interrelated with everyday life, the smart home has also been considered to improve the quality of life.

The idea of smart homes is connecting appliances together with the architecture of Internet of Things (IoT). The main application of smart homes can be classified but not limited to the following four categories [1,2,3].

1. Home control: home control is the most basic function that a home host can use as a central or remote controller to control smart devices.
2. Living condition optimization: The smart home system can optimize living conditions based on the data collected by sensors in terms of temperature, humidity, air quality, and so on.
3. Surveillance and security: smart home systems can protect the home physically by surveillance devices and smart door locks.
4. Digital entertainment: the entertainment system can connect all the devices into one Graphical User Interface (GUI) to apply a better entertainment experience.

**Figure 1:**
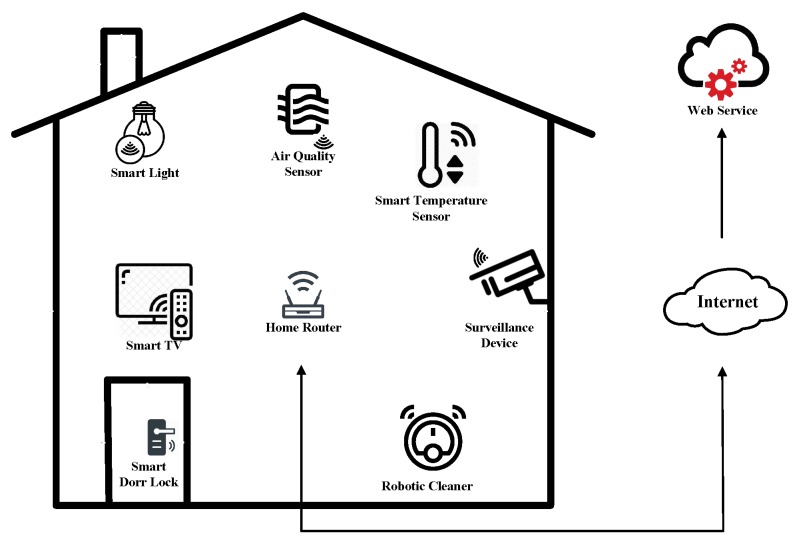
A smart home illustration.

We illustrate a smart home example in Figure 1. In this example, smart devices like smart light, air quantity sensor, smart temperature sensor, smart TV, surveillance device, smart door lock, and robotic cleaner are wirelessly connected to a home router, and the home router is wire connected with the Internet for more web services. The system model of the proposed protocol in this work is abstracted from this example.

### 1.1. Motivation

The tremendous application potential of smart homes has attracted the focus of academia and industry. However, another issue must be considered for better improvement of smart home, i.e., security issues [4,5]. Due to the wireless communication environment and the distributed network structure, smart home systems are facing a lot of attacks like Denial of Service (DoS) attack, black hole attack, Sybil attack, and so on.

To protect the smart home, two features of the sensors and devices in smart home should be considered. First, most of the sensors and devices are power and computation limited. Although such design enables optimization of the system’s power consumption, it leads to the fact that such nodes cannot operate large-scale computations. Second, the smart home system is designed with central authentication absent. Home routers can connect with all smart devices or nodes; however, the home router is not trustworthy enough for authentication. This case leads to the hard problem of key management in smart home. For the first problem, a lot of lightweight encryption [6,7,8,9] and lightweight authentication algorithms [10,11] have been proposed, but these algorithms are all based on a shared key which falls into the second problem. Thus, the existing security strategies are not suitable for smart homes and new directions should be considered.

Recently, physical layer security approach has attracted a lot of focus as an important supplement of traditional encryption-based security strategy. Moreover, the two main benefits of physical layer security are key needless and lightweight computation, which perfectly match the requirement of smart home security. Thus, it is desired to propose novel protocols based on physical layer security for smart home.

### 1.2. Related Work

**Smart Home Security.** The recent researches on smart home security are mainly focused on key management, device authentication, intrusion detection, and privacy preservation. For key management in smart homes, Reference [12] proposed lightweight session-key sharing in a smart home. Reference [13] designed the key establishment protocol considering power constraints. Also, in Reference [14], the authors considered key pairing for RF4CE ubiquitous smart homes. For device authentication, the authors in Reference [15] proposed a context-aware authentication for smart homes and the authors in Reference [16] proposed two-factor mutual authentication. For intrusion detection, Mehdi et al. investigated host-based intrusion detection in Reference [17] and Anthi et al. provided intrusion detection system with supervision for smart homes in Reference [18]. For privacy preservation, in Reference [19], a communication protocol has been proposed with a shared key by generating chaotic systems.

**Physical Layer Security.** The concept of physical layer security can be dated back to the basic research of Shannon in Reference [20], and Wyner generalized the concept in Reference [21] for practical use. The most relevant researches of physical layer security are those on the cooperative communications. Based on the credibility of the cooperative node, the physical layer security problem in cooperative communication can be divided into trusted relay security and untrusted relay security [22]. For the trusted relay security, the two-way relay usually conducts decode-and-forward to form a summed signal at the trusted relay [23,24]. By applying Multiple Inputs Multiple Outputs (MIMO) into cooperative communication, extra space redundancy will be used for securing transmission; see Reference [25] for distributed beamforming and Reference [26] for null space zero-forcing precoding. For untrusted relay security, Reference [27] reduced the obtained information at an untrusted relay with the help of a helper. Signal processing method is another way to resist the untrusted relay attack, the works in References [28,29] investigate the precoding design and the derivation of secrecy capacity for untrusted relay. Despite of the credibility of a relay node, another approach has been considered for securing two-way or multi-hop communication, i.e., physical layer network coding. Jayasinghe and coauthors focus on the secure beamforming for physical layer network coding for two-way relaying in Reference [30], and References [31,32] consider the multi-hop securing relaying with physical layer network coding.

### 1.3. Contributions

In this paper, we propose a novel secure group data exchange protocol in smart homes with physical layer network coding. The main contributions can be summarized as follows:

- We propose group data exchange protocols with a physical layer security approach. Each sensor node in a smart home conducts nested lattice physical layer network code, and summed coded data is formed in the home router. Such summed coded data cannot be divided into original data by an untrusted home router and external eavesdropper.
- We analyze the performance of our proposed protocol in terms of time slot cost, security resistance, and secrecy capacity. For the time slots cost, the proposed protocol is equal to the sensor node number. For security resistance, the summed coded data can prevent attacks from untrusted home routers as well as external eavesdroppers. For secrecy capacity, an expression has been derived for different attacks.
- We conduct simulations to demonstrate the theoretical analysis. Firstly, we show that the time slots cost of the proposed protocol is less than Time Division Multiple Access (TDMA) and network layer coding approach. Secondly, we perform the secrecy capacity with an untrusted home router attack. Thirdly, we perform the secrecy capacity with an external eavesdropper attack. Lastly, we perform the secrecy capacity with both an untrusted home router and an external eavesdropper attack.

### 1.4. Organization

The rest of this paper is organized as follows: Section 2 introduces the system model and basic conditions and definitions. Section 3 proposes the secure group data exchange protocol with physical layer network coding. Section 4 analyzes the performance of proposed protocol. Section 5 conducts simulations to perform. Finally, Section 6 concludes this work.

## 2. Preliminaries and System Model

In this section, we introduce the system model of the proposed information exchange protocol in a smart home. The basic conditions and definitions, transmission model, and security model are involved in these section sequentially.

### 2.1. System Model

**Figure 2:**
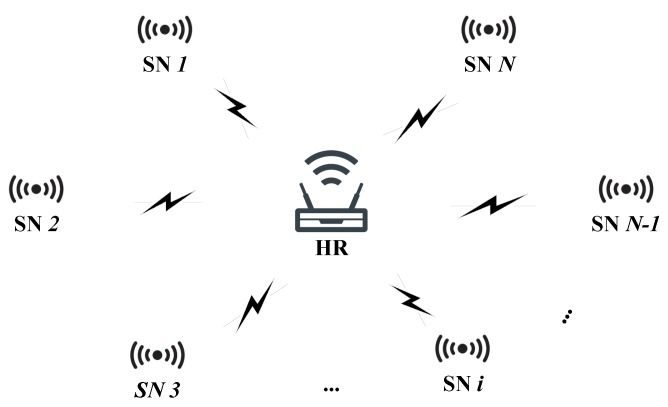
The system model for *N* sensor nodes data exchange in a smart home.

The proposed protocol considers the secure data exchange between different sensor nodes in a smart home. All the sensor nodes only directly connect with the home router, which is denoted as HR in the protocol. We abstracted the system model in Figure 2.

As the system model shown in Figure 2, we consider *N* sensor nodes in this work. The sensor node is shortened to SN in the protocol, and the *i*th sensor is denoted as $SN_{i}$. Each SN connects with HR with a wireless path. Note that we consider the situation that each SN can only communicate with HR, so there is no wire or wireless path between different SNs.

The proposed protocol involves *N* time slots. In the first time slot, which is denoted as an up-link phase, each SN send its data to be exchanged in the HR. The up-link phase is also called multiple access (MAC). The second time slot to the last time slot is denoted as a down-link phase. In this phase, HR broadcasts the collect data from the first time slot to all SNs. The down-link phase is also called broadcast (BC).

Actually, with such a transmission assumption, the HR acts as a relay in whole transmissions. In addition, HR works in the Decode and Forward (DF) model in the proposed protocol, so HR will first decode the collect data after the first time slot transmission and forward the data in the second time slot. We will have another assumption about HR that HR is equipped with multiple antennas. Such an assumption is practical for almost all the home routers on the market. We denote the antenna number of HR as $n_{HR}$ and we have the constraint as $n_{HR}\geq N-1$. Why must the antenna number satisfy this constraint? We will answer this question in the next section after the introducing of the up-link phase transmission.

We now discuss about the channel between HR and SN. We denote the up-link channel matrix between HR and $SN_{i}$ as $h_{i}$. Due to the reason that HR is equipped with multiple antennas, $h_{i}$ is a matrix if $SN_{i}$ is also equipped with multiple antennas. If $SN_{i}$ is only equipped with one antenna, $h_{i}$ is a vector. For now, most of the sensors in smart homes are single antenna, so we only use such a situation. We also denote the down-link channel matrix as $g_{i}$.

The SNs being equipped with a signal antenna is also the reason that N−1 time slots for the down-link phase are needed. For each, SN can only receive one data stream for one time slot; however, the HR has N−1 data streams that need to be broadcasted in the down-link phase. Such a situation can be easily explained with a detailed explanation of the protocol.

Finally, we discuss some definitions and notations in this paper. We use bold type in the equations to represent vectors and matrices and use normal type in the equations to represent scalars. Also, we use $\mathrm{Tr}\left(\cdot \right)$, ${\left(\cdot \right)}^{-1}$, $\det\left(\cdot \right)$, and $E\left(\cdot \right)$ to denote the trace, inverse or pseudo-inverse determinant, and expectation of matrix, respectively.

### 2.2. Transmission Model

We begin to formulate the transmission model of the proposed protocol. In the up-link phase, each SN sends its data to HR. We take SN *i* as an example; then, the data to be exchange is denoted as $d_{i}$. We assume the data is to be exchanged in binary field, so the elements of $d_{i}$ are 0 or 1. For the situation that the original data is not in binary field, the sensors convert them.

Before transmission, each SN conducts physical layer network coding for its own data:

(1) $$c_{i}=E\left(d_{i}\right)$$

where $E\left(\cdot \right)$ denote the physical layer network encoding. In this proposed protocol, we apply the best performance for now, i.e., nested lattice code, into the smart home.

After the physical layer network coding, precoding is conducted for all SNs for signal processing. The precoding vector is denoted as $P_{i}$, so the transmitting signal for SN *i* is as follows:

(2) $$X_{i}=P_{i}\cdot c_{i}$$

After the up-link phase transmission, the received signal at HR is as follows:

(3) $$Y_{HR}=\sum_{i=1}^{n}h_{i}X_{i}+z_{HR},$$

where $z_{HR}$ is the noise vector of HR and is modeled by $z_{HR}∼\mathrm{CN}\left(0,I_{n_{\mathrm{HR}}}\right)$.

With the received signal $Y_{HR}$, HR first conducts parallel to serial conversion for the vector and conducts decode for each element $Y_{HR}\left(i\right)$ as follows:

(4) $${\hat{Y}}_{HR}\left(i\right)=D\left(Y_{HR}\left(i\right)\right).$$

With the decoded ${\hat{Y}}_{HR}\left(i\right)$, HR forwards each element in each time slot in the down-link phase. In the second time slot, HR forwards ${\hat{Y}}_{HR}\left(1\right)$. Alike the up-link phase, precoding is also conducted for HR to generate the transmitting signal as follows:

(5) $$X_{HR_{2}}=P_{HR_{2}}\cdot {\hat{Y}}_{HR}\left(1\right).$$

The reason for the subscript 2 is that such a transmitting signal will be transmitted in the second time slot.

Then, HR broadcasts $X_{HR_{2}}$ to all SNs in the second time slot. We take SN*j* as an example; the received signal is as follows:

(6) $$Y_{j}=g_{j}\cdot X_{HR_{2}}+z_{j},$$

where $z_{j}$ is the noise at SN*j* modeled by $z_{j}∼\mathrm{CN}$$\left(0,1\right)$.

SN*j* conducts decoding for $Y_{j}$ to recover the data HR broadcast, and SN*j* stores the decoded data for the final recovering of all other data.

After decoding at the sensor node, the HR will begin the next time slot broadcasting. Identical with the second time slot, another round of precoding, broadcasting, recovering, and storing is conducted. The only part to be emphasized is that ${\hat{Y}}_{HR}\left(t-1\right)$ is broadcast in the *t*th time slot. HR broadcasts the last data ${\hat{Y}}_{HR}\left(N-1\right)$ in the *N*th time slot. After this time slot, each user recovers all other datum from all other sensors with the help of its own data. The detail of the data recover will be discussed in the next section.

### 2.3. Security Model

We consider two types of attacks in our proposed protocol. The first attack is called as untrusted HR attack, and the second attack is an external eavesdropping attack.

For the untrusted HR attack, we have a pessimistic assumption that the home router itself is an attacker. Such assumption exists because the home router and the sensors may be from different manufacturers. Then, the home router may try to collect data from the sensors and to send it back to its own manufacturer under the policy of the user experience improvement program. The funny thing is that the collecting and sending back of the datum is totally legal, and the users usually ignored such situations. Moreover, almost all existing data exchange protocols cannot prevent the home router from reaching the data; thus, it is desired to consider a protocol to prevent the eavesdropping from the home router in the physical layer.

The external eavesdropper attack comes from two situations. First, the sensors which are not involved in the data exchange: The sensors in each round of data exchange are different, and the sensors which are not involved in this round may try to recover the exchanged data. Second, the sensors or home routers from other homes are also able to wiretap the channel due to the wireless communication environments. These sensors or router cannot be prevented with authentication, so we may try to prevent them in the physical layer.

### 2.4. Basic Conceptions and Notations for Nested Lattice Code

As in the literature review, nested lattice code has been shown to be the most efficient and reliable coding algorithm for now. Thus, we choose to use nested lattice code in the proposed protocol. Thus, in this subsection, we briefly introduce some basic conceptions and notations for nested lattice code.

**Definition** **1.** **Lattice** Λ: *An n dimension lattice is a discrete subgroup of $R^{n}$; it is the linear combinations of some basis vectors:*

(7) $$\Lambda =\left\{\lambda =xG_{\Lambda }:x\in Z^{n}\right\},$$

*where $G_{\Lambda }$ is called generator matrix for lattice* Λ *by defining as follows:*

(8) $$G≜{\left[g_{1}^{t}\left|\cdots \right|g_{n}^{t}\right]}^{t}.$$

**Definition** **2.** **Quantizer $Q_{\Lambda }\left(\cdot \right)$** *: The quantizer function of lattice is the mapping $Q_{\Lambda }\left(\cdot \right):R^{n}\to \Lambda$, i.e., mapping a vector to the closet lattice point as follows:*

(9) $$Q_{\Lambda }\left(x\right)=arg\begin{array}{c} \underbrace{min}\\ \lambda \in \Lambda \end{array}∥x-\lambda ∥,$$

*where $∥\cdot ∥$ represents the Euclidean norm.*

**Definition** **3.** **Fundamental Region $R_{\Lambda }$** *: $R_{\Lambda }$ is the Voronoi Region of the original, and Voronoi Region is defined as follows:*

(10) $$V_{\Lambda }\left(\lambda \right)≜\left\{x:Q_{\Lambda }\left(x\right)=\lambda \right\},$$

*which is the closest points set of a lattice point.*

*Thus, the fundamental region is the following:*

(11) $$R_{\Lambda }=\left\{x:Q_{\Lambda }\left(x\right)=0\right\}$$

**Definition** **4.** **Modulo-Λ***: The modulo-*Λ *operation is defined as follows:*

(12) $$x\;\mathrm{mod}\;\Lambda ≜x-Q_{\Lambda }\left(x\right)$$

**Definition** **5.** **Nested Lattices $\left(\Lambda_{f},\Lambda_{c}\right)$** *: If $\Lambda_{f}$ is a lattice itself and $\Lambda_{c}$ is a sublattice of $\Lambda_{f}$, i.e., $\Lambda_{c}\subseteq \Lambda_{f}$, then the lattice pair $\left(\Lambda_{f},\Lambda_{c}\right)$ is called nested. Under such case, $\Lambda_{f}$ is called fine lattice and $\Lambda_{c}$ is called coarse lattice.*

**Definition** **6.** **Nested Lattice Code C** *: The nested lattice codebook C is defined as all the coset leaders in $\Lambda_{f}/\Lambda_{c}$ as follows:*

(13) $$C\left(\Lambda_{c},\Lambda_{f}\right)=\Lambda_{f}\;\mathrm{mod}\;\Lambda_{c}.$$

*Geometrically speaking, codeword $C\left(\Lambda_{c},\Lambda_{f}\right)$ is the lattice point of $\Lambda_{f}$ and lies in the fundamental region of $\Lambda_{c}$:*

(14) $$C=\{\Lambda_{f}\cap R_{\Lambda_{c}}\}.$$

**Figure 3:**
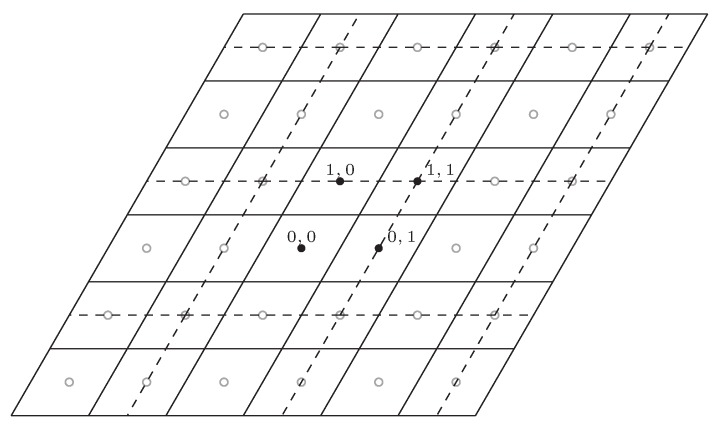
Nested lattice code with $g_{1}=\left(1,0\right)$, $g_{2}=\left(1/2,\sqrt{3}/2\right)$, and $\Lambda_{c}=2\Lambda_{f}$.

**Example** **1.** *We give an example of nested lattice code. The fine lattice $\Lambda_{f}$ is generated by $g_{1}=\left(1,0\right)$ and $g_{2}=\left(1/2,\sqrt{3}/2\right)$. The coarse lattice is $\Lambda_{c}=2\Lambda_{f}$. We use Figure 3 to illustrate this example. In the figure, the Voronoi region of fine lattice is represented by a full line and the the Voronoi region of coarse lattice is represented by a dashed line. The four codewords of nested lattice is represented by the solid dots, and the other lattice point of fine lattice is represented by the soft dots.*

## 3. The Proposed Data Exchange Protocol

In this section, we will introduce the proposed secure group data exchange protocol in a smart home with physical layer network coding. According to the system model, the discussion on the protocol is also divided into three parts: up-link phase, decode-and-forward, and down-link phase.

### 3.1. Up-Link Phase

In the up-link phase, all SNs send their data to HR at the same time. Before transmitting, each SN conducts physical layer network coding for secure, reliable, and efficient transmission. As in the literature review, nested lattice code has been shown to be the most efficient and reliable coding algorithm for now. Thus, we choose to use nested lattice code in the proposed protocol. The basic introduction of nested lattice code has been discussed in the last section, and we denote the nested lattice code as $C=\left\{c_{1},c_{2},\cdots ,c_{i},\cdots \right\}$. The encoding could be rewritten as follows:

(15) $$E\left(\cdot \right):d_{i}\in \to c_{i}.$$

After the encoding, each SN conducts precoding $P_{i}$ to generate $n_{HR}$ equivalent parallelized subchannels. The signal processing algorithm could be referred to in References [28,29,33], especially the work in Reference [33] presents the group information exchange. In this work, the neighbour nodes align its signals into the same subchannel. Similar to this work, the data from $SN_{i}$ and $SN_{N-1}$ and from $SN_{i}$ and $SN_{N+1}$ are aligned into the same subchannels. Especially, $SN_{1}$ only aligns its data with $SN_{2}$ in the first subchannel and $SN_{N}$ only aligns its data with $SN_{N-1}$ in the last subchannel. We focus the physical layer network coding in this work, so we will not go into detail on the design of $P_{i}$.

Before we introduce the decode and forward in HR, we recall the question of the antenna number constraint $n_{HR}\geq N+1$. Due to each SN aligning its data with its neighbour SN for two times and $SN_{1}$ and $SN_{N}$ only aligning one time, at least N−1 subchannels are required. To provide at least N−1 independent data streams, HR must have at least N−1 antennas.

### 3.2. Decode and Forward in HR

After the up-link phase transmission, HR receives the converged data from all SNs. The precoding matrices reduce the wireless channel interference, generate N−1 subchannels, and align the coded data from two neighbour SNs into the same subchannel. Thus, the received signal at HR could be written as follows:

(16) $$\begin{array}{cc} Y_{HR}= & \sum_{i=1}^{n}h_{i}X_{i}+z_{HR}\\ = & \left[\begin{array}{c} \begin{array}{cc} c_{1} & ⊕c_{2}\\ c_{2} & ⊕c_{3}\\ \; & \cdots \\ c_{i-1} & ⊕c_{i}\\ c_{i} & ⊕c_{i+1}\\ \; & \cdots \\ c_{N-1} & ⊕c_{N} \end{array} \end{array}\right] \end{array}+z_{HR}$$

$Y_{HR}$ is an N−1×1 vector, and we conduct parallel to serial conversion to convert this vector into N−1 elements as follows:

(17) $$Y_{HR}\left(i\right)=\left[c_{i}⊕c_{i+1}\right]+z_{HR}\left(i\right).$$

Then, the nested lattice code decoding is conducted for each element to recover the sum code of two neighbour SNs. By the nested lattice decoding, $Y_{HR}\left(i\right)$ is sent by nearest point quantizer as follows:

(18) $$\begin{array}{ccc} {\hat{Y}}_{HR}\left(i\right)= & Y_{HR} & \;\mathrm{mod}\;\Lambda_{c}\\ = & \left[c_{i}⊕c_{i+1}\right]+z_{HR}\left(i\right) & \;\mathrm{mod}\;\Lambda_{c}. \end{array}$$

To be noted, the decoded data is still a codeword in $\left(\Lambda_{f},\Lambda_{c}\right)$ and HR will broadcast the decoded words in the down-link phase. Before that, we give an example to show the decode at HR.

**Figure 4:**
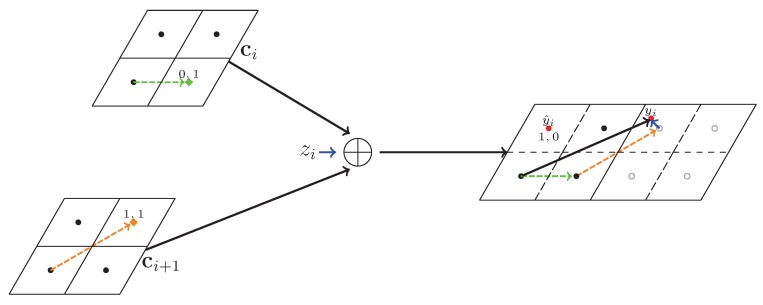
Nested lattice decode example.

**Example** **2.** *We follow Example 1, i.e., the fine lattice $\Lambda_{f}$ is generated by $g_{1}=\left(1,0\right)$ and $g_{2}=\left(1/2,\sqrt{3}/2\right)$ and the coarse lattice is $\Lambda_{c}=2\Lambda_{f}$. The sent code at $SN_{i}$ is 01, which is shown as a green diamond in Figure 4, and the sent code at $SN_{i+1}$ is 11, which is shown as an orange diamond. The noise is shown as a blue arrow, and the nearest point quantizer sent $y_{i}$ back to codeword as ${\hat{y}}_{i}$ and is shown as a red dot.*

### 3.3. Down-link Phase

After decoding, HR broadcasts the decoded word in the down-link phase. Due to the reason that SN is only equipped with a single antenna, HR can only broadcast one summed codeword in each time slot. Recalling that there are $N_{1}$ summed codewords, the down-link phase needs N−1 time slots to accomplish the broadcasting.

The up-link phase costs one time slot; the down-link phase begins in the second time slot. In this time slot, HR broadcasts the first element of the decoded word, i.e., ${\hat{Y}}_{HR}=c_{1}⊕c_{2}$. Similarly, in the *t*th time slot, HR broadcasts the $\left(t-1\right)$th element of the decoded word as ${\hat{Y}}_{HR}=c_{t-1}⊕c_{t}$.

The down-link precoding is also conducted for each time slot; however, we still avoid going into detail on the precoding design. The precoding at HR and filtering at each SN can successfully reduce the channel interference. Then, in the *t*th time slot, each SN applies nested lattice decoding to obtain $c_{t-1}⊕c_{t}$. After N−1 times broadcasting, each SN recovers all elements of the summed coded data. With these summed coded data, each SN recovers the original data from all other nodes with the help of its own data.

The recovering progress is also known as successive decoding algorithm. Taking $SN_{i}$ as an example, $SN_{i}$ receive $c_{i-1}⊕c_{i}$ in the *t*th time slot. With the help of its own coded data $c_{i}$, $SN_{i}$ recovers $c_{i-1}$ and decodes it back to binary field. Identically, $SN_{i}$ recovers $c_{i-2}$ with the help of $c_{i-1}$ and successively recovers $c_{i-3}$ and $c_{i-4}$ until $c_{1}$. In the t+1th time slot, $SN_{i}$ receives $c_{i}⊕c_{i+1}$ and $SN_{i}$ recovers $c_{i+1}$. Then, in the t+2th time slot, $SN_{i}$ recovers $c_{i+2}$ with the help of $c_{i+1}$. Identically, $SN_{i}$ recovers $c_{i+3}$ and $c_{i+4}$ until $c_{N}$ in the last time slot.

## 4. Performance Analysis for Proposed Protocol

In this section, we evaluate the performance of the proposed data exchange protocol. Three types of analyses have been conducted in terms of time slots cost, security resistant, and secrecy capacity.

### 4.1. Time Slots Cost Analysis

With the introduction of the proposed protocol, we can clearly see that the time slots cost of the protocol is *N*. We denote the time slots cost as $TS_{PHY}$, so we have $TS_{PHY}=N$. For comparison, we also give the time slots cost of the naive TDMA protocol and network layer coding protocol for *N* SN data exchanges.

For the naive TDMA, only one SN communicates with HR in each time slot. Thus, it takes 4 time slots for 2 SNs to accomplish data exchange. In total, the time slots cost can be computed as follows:

(19) $$TS_{TDMA}=4*C_{N}^{2}=2N*\left(N-1\right).$$

For network layer coding, it prefers opposite progress to the proposed protocol. In the up-link phase, each SN sends its data to HR from the first time slot to the *N*th time slot. HR stores all the data, conducts network layer coding after the *N*th time slot, and broadcasts the coded data in the N+1th time slot. Thus, the total time slots cost is $TS_{NET}=N+1$.

Apparently, the proposed protocol takes the least time slots cost for the same data exchange application. We will illustrate the numerical figure of this result in the next section, and we will show that the TDMA approach is not available when the SNs number is large.

### 4.2. Security-Resistant Analysis

Recalling the security model, two types of attacks have been considered, i.e., untrusted HR attack as well as external eavesdropping attack.

#### 4.2.1. Untrusted HR Attack

As in the description in Section 2.3, HR can act two roles in a smart home. Usually, HR acts as the helper to assist the sensor nodes; however, HR can also act as a potential attacker to collect data from the sensors for improper usage.

For the proposed protocol, HR is the receiver for the up-link phase, so HR can distinguish the subchannels and the corresponding data in each subchannel. However, the received data at HR is a summed codeword of two neighbour SNs, and HR cannot directly recover the individual codewords from each SN.

We use the example in Section 3.2 to explain why the HR cannot recover the original data. The received data ${\hat{y}}_{i}$ at HR is the summed codeword of 01 from $SN_{i}$ and 11 from $SN_{i+1}$. However, ${\hat{y}}_{i}$ can also be the sum of 11 from $SN_{i}$ and 01 from $SN_{i+1}$. Also, it could be the sum of 00 and 10 or 10 and 00. In other words, for the received codeword ${\hat{y}}_{i}$, it could be any original data. Thus, HR cannot tell which data SN has sent.

#### 4.2.2. External Eavesdropping Attack

Before we analyze the security performance at the external eavesdropper, we first discuss the wiretap channel. Although the eavesdropping may come from two types of attackers, i.e., in home sensor and out home sensor, the situation is totally identical. Thus, we consider these two attackers as one type called Eve, and $Eve_{i}$ is the eavesdropper between $SN_{i}$ and HR in the up-link phase.

We denote the wiretap channel matrix between $SN_{i}$, and $Eve_{i}$ is $h_{ie}$, so the received signal at $Eve_{i}$ is as follows:

(20) $$\begin{array}{cc} Y_{E_{i}}= & \sum_{i=1}^{N}h_{ie}X_{i}+z_{E_{i}}\\ = & \sum_{i=1}^{N}h_{ie}P_{i}c_{i}+z_{E_{i}}. \end{array}$$

For Equation (20), the precoding matrices $P_{i}$ can reduce the channel interference $h_{i}$; however, only the situation $h_{ie}=h_{i}$$Eve_{i}$ can correctly recover the summed code. Otherwise, each eavesdropper can only receive some superimposed signals. Moreover, even the eavesdropper is very close to the HR and the channel is identical to HR. The eavesdropper can only recover the summed codeword, which cannot be separated from the original data.

For the down-link phase eavesdroppers, they cannot launch more effective attacks than in the up-link phase. Due to the evidence of data processing, the mutual information between the down-link phase Eve cannot be larger than the up-link phase. Thus, the down-link phase eavesdroppers cannot launch more effective attacks.

### 4.3. Secrecy Capacity Analysis

The secrecy capacity is the most general and important performance metric for physical layer security protocol. With different attack models, the analysis is in terms of untrusted HR, external Eve, and both HR and Eve.

#### 4.3.1. Secrecy Capacity with Untrusted HR

We first derive the secrecy capacity with only untrusted HR attacks. In this case, each SN regulates its transmission rate $R_{iUH}$ to avoid HR obtaining enough information to correctly recover any information. Thus, the secrecy capacity under this case is as follows:

(21) $$C_{s_{UH}}=\frac{1}{N}{\left[\sum_{i=1}^{N}R_{i_{UH}}-R_{H_{UH}}\right]}^{+},$$

where $R_{UH}$ is the obtained information at an untrusted HR. The coefficient $\frac{1}{N}$ is because it takes *N* time slots to accomplish the whole data exchange.

Such an expression for secrecy capacity is a general derivation, and we must explore the details of $R_{i_{UH}}$ and $R_{UH}$ to obtain the final result. The $R_{i_{UH}}$ of the proposed protocol is as follows:

(22) $$R_{i_{UH}}=\log\;\det\left(I+h_{i}Q_{i}h_{i}^{t}\right),$$

where $Q_{i}$ is the input covariances of $SN_{i}$:

(23) $$Q_{i}=E\left(X_{i}X_{i}^{t}\right)=E\left(P_{i}c_{i}c_{i}^{t}P_{i}^{t}\right)=E\left(P_{i}P_{i}^{t}\right).$$

The third step can be obtained because the codeword is independent from each other.

For $R_{H_{UH}}$, it is the mutual information between HR and all SNs, so we have the following:

(24) $$\begin{array}{cc} R_{H_{UH}}= & I\left(Y_{HR};X_{1},X_{2},\cdots ,X_{N}\right)\\ = & \log\;\det\left(I+\sum_{i=1}^{N}h_{i}Q_{i}h_{i}^{t}\right). \end{array}$$

With Equations (22) and (24), Equation (21) can be rewritten as follows:

(25) $$\begin{array}{cc} C_{s_{UH}}= & \frac{1}{N}{\left[\sum_{i=1}^{N}R_{i_{UH}}-R_{H_{UH}}\right]}^{+}\\ = & \frac{1}{N}\log\;\det\left[\frac{\prod_{i=1}^{N}\left(I+h_{i}Q_{i}h_{i}^{t}\right)}{I+\sum_{i=1}^{N}h_{i}Q_{i}h_{i}^{t}}\right]. \end{array}$$

#### 4.3.2. Secrecy Capacity with External Eve

Under this case, the HR is not an attacker; however, external eavesdroppers exist to wiretap the channel. As in the aforementioned description, we only consider the eavesdroppers for the up-link phase as the down-link phase eavesdroppers cannot launch more effective attacks. In the up-link phase, each SN, HR, and the eavesdropper between them forms a classical wiretap channel. Thus, the secrecy capacity is the sum of the secrecy capacity for each wiretap subchannel. Then, we have the following:

(26) $$C_{s_{EE}}=\frac{1}{N}{\left[\sum_{i=1}^{N}\left(R_{i_{EE}}-R_{{Ei}_{EE}}\right)\right]}^{+},$$

where $R_{i_{EE}}$ is the transmission rate of user *i* for an external eavesdropper attack and its analysis is identical to Equation (22) as follows:

(27) $$R_{i_{EE}}=\log\;\det\left(I+h_{i}Q_{i}h_{i}^{t}\right).$$

$R_{{Ei}_{EE}}$ is the obtained information of the external eavesdropper. Consider the situation that eavesdroppers may not wiretap some SN; we use the variable $\nu_{i}$ to describe whether an eavesdropper exists. If an eavesdropper wiretaps $SN_{i}$, we have $\nu_{i}=1$; otherwise, $\nu_{i}=0$. Then, we give the expression of $R_{{Ei}_{EE}}$ as follows:

(28) $$\begin{array}{cc} R_{Ei_{EE}}= & I\left(Y_{E_{i}};X_{i}\right)\\ = & \log\;\det\left(I+\nu h_{ie}Q_{i}h_{ie}^{t}\right). \end{array}$$

With Equations (27) and (28), Equation (26) can be rewritten as follows:

(29) $$\begin{array}{cc} C_{s_{EE}}= & \frac{1}{N}{\left[\sum_{i=1}^{N}\left(R_{i_{EE}}-R_{{Ei}_{EE}}\right)\right]}^{+}\\ = & \frac{1}{N}\log\;\det\left[\prod_{i=1}^{N}\left(\frac{I+h_{i}Q_{i}h_{i}^{t}}{I+\nu h_{ie}Q_{i}h_{ie}^{t}}\right)\right]. \end{array}$$

#### 4.3.3. Secrecy Capacity with Both Attacks

In this last section, we consider the most pessimistic situation that the HR is untrusted and that there are external eavesdroppers. The analysis for such a situation is a composition of the former two cases, so we have the following:

(30) $$C_{s_{UE}}=\frac{1}{N}{\left[\sum_{i=1}^{N}\left(R_{i_{UE}}-R_{Ei_{UE}}\right)-R_{H_{UE}}\right]}^{+}.$$

The analyses of $R_{i_{UE}}$, $R_{Ei_{UE}}$, and $R_{H_{UE}}$ are identical to the former two cases, so we have the following:

(31) $$R_{i_{UE}}=\log\;\det\left(I+h_{i}Q_{i}h_{i}^{t}\right),$$

(32) $$\begin{array}{cc} R_{Ei_{UE}}= & I\left(Y_{E_{i}};X_{i}\right)\\ = & \log\;\det\left(I+\nu h_{ie}Q_{i}H_{ie}^{t}\right), \end{array}$$

(33) $$\begin{array}{cc} R_{H_{UE}}= & I\left(Y_{HR};X_{1},X_{2}\cdots X_{N}\right)\\ = & \log\;\det\left(I+\sum_{i=1}^{N}h_{i}Q_{i}h_{i}^{t}\right). \end{array}$$

With Equations (31), (32), and (33), Equation (30) can be written as follows:

(34) $$\begin{array}{cc} C_{s_{UE}}= & \frac{1}{N}{\left[\sum_{i=1}^{N}\left(R_{i_{UE}}-R_{Ei_{UE}}\right)-R_{H_{UE}}\right]}^{+}\\ = & \frac{1}{2}\log\;\det\left[\frac{\prod_{i=1}^{N}\left(\frac{I+h_{i}Q_{i}h_{i}^{t}}{I+\nu h_{ie}Q_{i}h_{ie}^{t}}\right)}{I+\sum_{i=1}^{N}h_{i}Q_{i}h_{i}^{t}}\right]. \end{array}$$

## 5. Simulation Results

In this section, simulations have been conducted to perform the proposed protocol in terms of time slots cost, secrecy capacity with an untrusted HR attack, secrecy capacity with an external eavesdropper, and secrecy capacity with an untrusted HR and an external eavesdropper attack.

**Figure 5:**
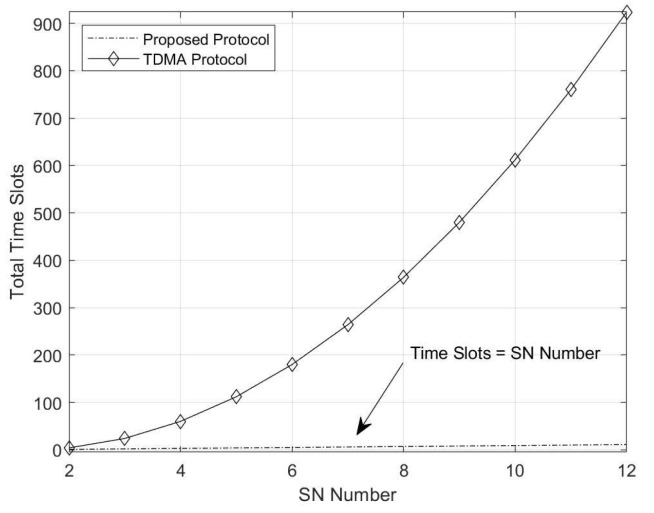
Time slots cost comparison between the TDMA protocol and the proposed protocol.

**Figure 6:**
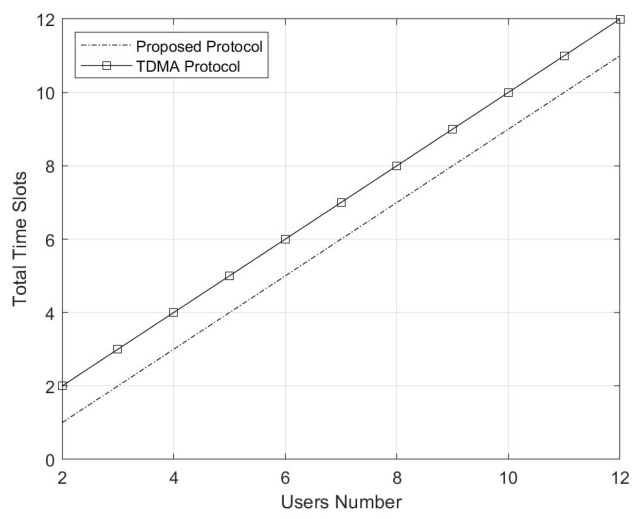
Time slots cost comparison between the network code protocol and the proposed protocol.

Following the analysis in Section 4.1, we first illustrate the time slots cost in Figure 5 and Figure 6. We first compare the time slots cost between the naive TDMA approach and our proposed protocol in Figure 5. The results demonstrate the analysis that the time slots cost of TDMA is tremendous when the SN number is over 4. Although the time slots cost of our protocol is also increasing with the increase in SN number, the cost is still acceptable.

We also compare the time slots cost between the network code protocol and our proposed protocol. The time slots cost difference of these two approaches is always 1 whether the SN number increases or not. Thus, the result in Figure 6 is two parallel lines with a gap of 1.

In the following, we show the numerical results of secrecy capacity of the proposed protocol. For the later simulations, the SN is set as 5 and the antenna number of HR is 4. In each round, we generate new channels between SN and HR and with the channel between SN and Eve. The final results obtained over 10,000 rounds of iteration.

**Figure 7:**
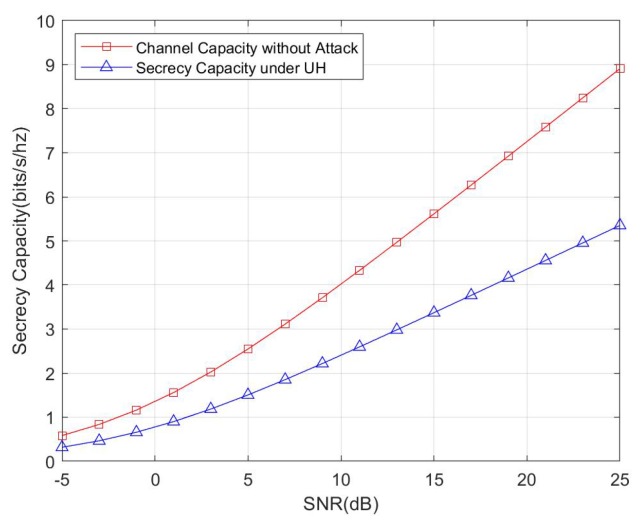
Comparison between secrecy capacity under an untrusted home router (HR) and channel capacity without attack.

We first compare the secrecy capacity under an untrusted HR attack with channel capacity without any attacks, and the result is shown in Figure 7. Apparently the secrecy capacity is less than the channel capacity without an attack, which is in accordance with the evidence that we sacrificed the transmission recourse for improving the security.

**Figure 8:**
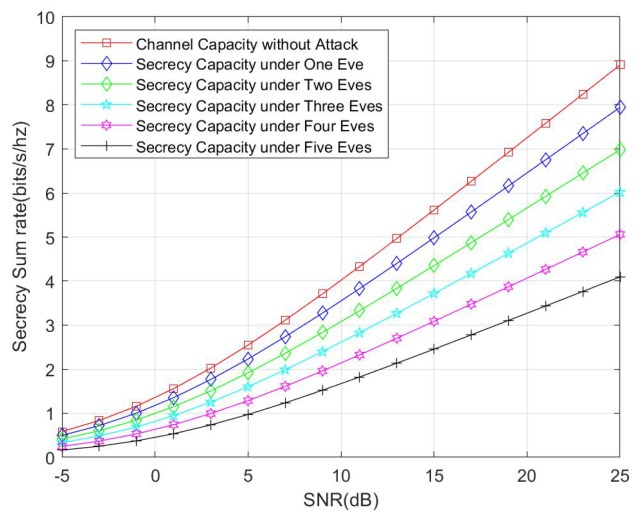
Comparison between secrecy capacity under an external eavesdropper and channel capacity without attack.

We also compare the secrecy capacity under an external eavesdropper with channel capacity without any attacks. The simulation result is shown in Figure 8. We consider the cases of one eavesdropper, two eavesdroppers, three eavesdroppers, four eavesdroppers, and five eavesdroppers. According with theoretical analysis, the secrecy capacity is reduced with the increasing number of eavesdroppers. Another interesting fact that should be noticed is that, when comparing Figure 8 with Figure 7, the secrecy capacity under an untrusted HR attack is less than the secrecy capacity under one external eavesdropper, and this fact also reflects that the untrusted HR can launch more effective attacks than a single eavesdropper.

**Figure 9:**
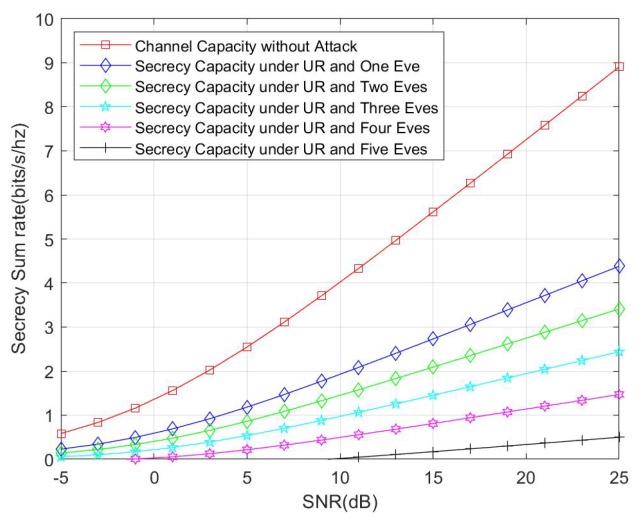
Comparison between secrecy capacity under an untrusted home router along with an external eavesdropper and channel capacity without attack.

Finally, we compare the secrecy capacity under both untrusted HR and external eavesdropper attack with channel capacity without any attacks. The result is illustrated in Figure 9. Similar to the former simulation, we also consider the number of eavesdroppers from one to five. The result shows that the secrecy capacity is only half of the channel capacity for only one eavesdropper. For the worst case, i.e., five eavesdropper, an outage occurred when the channel situation was very bad.

## 6. Conclusions

As one of the main drawbacks of smart homes, security issues should not be neglected for further application of Internet of Things. To address this problem, we propose a novel secure group data exchange protocol in this paper for smart homes with a physical layer security approach. As the core technique to obtain secure data exchange, physical layer network coding is applied for smart homes. Nested lattice code is carried out at each sensor node, and summed coded data is formed in a home router. With such summed coded data, attacks can be prevented from both an untrusted home router as well as an external eavesdropper. We analyzed the proposed protocol in terms of time slots cost, security resistance, and secrecy capacity. Finally, we conducted simulations to demonstrate the theoretical analysis.

The future work of the proposed protocol includes the following:

1. The optimization of secrecy capacity, especially with strict power constraints of sensor nodes.
2. The design of a novel physical layer network coding algorithm with less computation cost.
3. The implementation of the proposed protocol into real smart home systems.
